# Chromium propionate supplementation alters animal growth performance, carcass characteristics, and skeletal muscle properties in feedlot steers

**DOI:** 10.1093/tas/txaa146

**Published:** 2020-07-30

**Authors:** Jessica O Baggerman, Zachary K Smith, Alex J Thompson, Jongkyoo Kim, Jerilyn E Hergenreder, Whitney Rounds, Bradley J Johnson

**Affiliations:** 1 Department of Animal and Food Sciences, Texas Tech University, Lubbock, TX; 2 Kemin Industries, Inc., Des Moines, IA

**Keywords:** beef cattle, biopsy, GLUT4, skeletal muscle

## Abstract

The objective of this study was to evaluate the effects of increasing concentrations of Cr propionate (CrP) on feedlot performance, blood parameters, carcass characteristics, and skeletal muscle fiber properties in feedlot steers. Crossbred steers (*n* = 32; 367 ± 2.5 kg; 16 pens; 2 hd/pen) were blocked by body weight (BW), and treatment was randomly assigned to pen: (1) 0 mg added Cr/kg diet dry matter (DM) (control), (2) 0.15 mg added Cr/kg diet DM (CrP; KemTRACE Chromium 0.04%, Kemin Industries, Des Moines, IA), (3) 0.30 mg added Cr/kg diet DM, and (4) 0.45 mg added Cr/kg diet DM. Steers were fed ad libitum, and the treatment was top-dressed at the time of feeding. Body weights, blood samples, and longissimus biopsies were collected before feeding on days 0, 28, 56, 91, 119, and 147. Blood sera were harvested for analysis of glucose, insulin, sera urea nitrogen, and non-esterified fatty acid concentrations. Longissimus biopsies were collected for gene expression, protein expression, and immunohistochemical (IHC) analysis. Pen was the experimental unit for live and carcass data, and steer was the experimental unit with day as a repeated measure for sera and IHC analyses. For the entire duration of the trial, a linear increase in average daily gain (ADG) (*P* = 0.01) and improvement in G:F was observed (*P* = 0.01) with no change in DMI (*P* = 0.11) with increasing CrP. A linear increase in hot carcass weight (HCW) (*P* ≤ 0.01) with no other changes in carcass composition were noted (*P* ≥ 0.38) as the level of dietary CrP increased. There was no effect of treatment on any sera parameters measured (*P* ≥ 0.10). No difference was detected for gene or protein expression of glucose transporter type 4 (GLUT4) due to CrP supplementation (*P* ≥ 0.10). For skeletal muscle fiber distribution and cross-sectional area, there was no effect of treatment (*P* ≥ 0.10). Density of total GLUT4 did not change due to CrP (*P* ≥ 0.10). Internalization of GLUT4 was increased in the 0.30 and 0.45 mg/kg treatments (*P* < 0.01). For total nuclei density and myonuclei density, there were treatment × day interaction tendencies (*P* ≤ 0.08). Supplementation of CrP did not alter density of satellite cells (*P* ≥ 0.10). The number of transporters located in the sarcolemma of skeletal muscle fibers did decrease, implying fewer proteins were needed to transport extracellular glucose into the muscle fiber. Therefore, CrP may augment cellular function and growth via increased efficiency of GLUT4 function. These results indicated CrP increases BW, ADG, and HCW, without changes in circulating sera parameters or total GLUT4 expression.

## INTRODUCTION

Chromium (Cr) was identified as an essential trace mineral for mammalian species due to increased glucose tolerance during addition of Cr^3+^ (Schwarz and Mertz, 1959). The only US FDA approved form of Cr which can be supplemented to cattle is Cr propionate (CrP; Kemin Industries, Inc., Des Moines, IA), which can be fed up to 0.50 mg/kg in complete mixed rations. Addition of either Cr-l-methionine or CrP improved glucose tolerance in feedlot cattle subjected to a glucose tolerance test (Kegley et al., 2000; Spears et al., 2012). Additionally, a positive linear response in average daily gain (ADG) was observed when steers were supplemented CrP up to 0.30 mg/kg, as well as improvement in immune parameters following an endotoxin challenge (Bernhard et al., 2012). Several studies have shown improved live performance and carcass characteristics in cattle supplemented with various forms of Cr for numerous duration (Pollard et al., 2002; Barajas et al., 2008a, 2008b; Kneeskern et al., 2016). Glucose transporter type 4 (GLUT4) is the most prevalent glucose transporter for skeletal muscle as evidenced by the knockout of GLUT4 leading to increased insulin resistance and intolerance of glucose (Zisman et al., 2000). Impacts of supplementation of organic Cr sources for performance in feedlot cattle have reported mixed results and seems to be influenced by age, level of concentrate feeding, and level of adiposity (Pollard et al., 2002; Sánchez-Mendoza et al., 2014; Sung et al., 2015; Kneeskern et al., 2016; Van Bibber-Krueger et al., 2016). Previous studies have only supplemented CrP up to 0.30 mg/kg of the total mixed ration, and the effect of CrP on skeletal muscle growth and metabolism is not completely understood. Therefore, the objective of this study was to measure changes in live performance, carcass characteristics, sera parameters, skeletal muscle fiber distribution, cross-sectional area, satellite cell populations, and GLUT4 receptor characteristics of feedlot steers receiving increasing levels of CrP, approaching the US legal limit of 0.50 mg/kg to the diet on a dry matter (DM) basis.

## MATERIALS AND METHODS

The experimental protocol was approved by Texas Tech University Animal Care and Use Committee (#14067). The study was conducted at the Texas Tech University Burnett Center located near New Deal, TX.

### Animals

Continental crossbred steers (*n* = 32; 367 ± 2.5 kg) were obtained from a single source and processed after arrival at the Burnett Center. Steers were vaccinated against viral respiratory diseases, *Mycoplasma bovis*, and clostridial diseases (Vista 5 SQ, Merck Animal Health, Summit, NJ; Vision CD, Merck Animal Health; Myco-Bac B, Texas Vet Lab, Inc., San Angelo, TX), treated for internal and external parasites (Dectomax Pour-On solution, Zoetis USA, Florham Park, NJ) according to label directions, and given an unique individual ear tag for identification. Steers were transitioned from the step-up ration to a final finishing ration (Table 1) on day 7, and both were formulated to meet or exceed dietary requirements described by the NRC (2000). The ration was mixed daily, and steers were fed once daily at 0800 hours to ensure ad libitum access to feed throughout the feeding trial using a slick bunk management approach. Orts were observed daily, and feed calls were adjusted to target ~0.45 kg DM orts remaining prior to next feed delivery. Feed was delivered to each pen via a tractor-pulled mixer (Roto-Mix 84-8, Dodge City, KS). Steers were implanted on days 0 and 88 using Synovex Choice (14 mg estradiol benzoate and 100 mg trenbolone acetate; Zoetis). Beginning on day 119, all steers received 300 mg·steer^−1^·d^−1^ of ractopamine HCl (Optaflexx, Elanco Animal Health, Greenfield, IN) mixed into the basal ration until the conclusion of the experiment. Animals were monitored daily for signs of clinical illness or lameness. If an animal was found to need further treatment, it was maintained in a hospital pen. If the steer was returned to their home pen, that feed remained credited to the home pen. If a steer was removed from the study, the feed delivered to the hospital pen was deducted from the pen up until the point of hospitalization.

**Table 1.** Description of experimental diets^1^

Ingredients^2^, % DM	Step-up^3^	Final^3^
Steam-flaked corn	67.85	75.10
Alfalfa hay, chopped	14.64	10.38
Cottonseed meal	7.00	4.97
Molasses	3.71	2.94
TTU mineral supplement^4^	2.00	2.29
Fat (yellow grease)	3.01	2.43
Urea	0.89	0.97
Calcium carbonate	0.90	0.91
Nutritional profile, DM^5^		
DM, %	83.5	81.3
Crude protein, %	14.8	14.9
Acid detergent fiber, %	10.8	8.7
Neutral detergent fiber, %	16.1	14.4
Ash, %	4.6	4.4
Total digestible nutrients, %	87.7	89.9
NE_M_, Mcal/kg	2.18	2.24
NE_G_, Mcal/kg	1.50	1.55

^1^Diets were formulated to meet or exceed NRC (2000) requirements for growing–finishing beef cattle.

^2^Treatments were added to the basal ration at the rate of 0.227 g/hd daily via a ground-corn-based top-dress mixture.

^3^Step-up ration was fed until day 7. Final ration was fed starting on day 8 through remainder of experiment.

^4^Supplement composition (DM basis): 66.476% cottonseed meal; 15.000% salt; 10.000% potassium chloride; 4.167% ammonium sulfate; 0.986% zinc sulfate; 0.750% Rumensin (176.4 mg/kg; Elanco Animal Health, Indianapolis, IN); 0.648% dicalcium phosphate; 0.563% Tylan (88.2 mg/kg; Elanco Animal Health); 0.500% ENDOX (Kemin Industries, Des Moines, IA); 0.333% manganese oxide; 0.196% copper sulfate; 0.158% vitamin E (500 IU/g; DSM Nutritional Products, Inc.); 0.125% selenium premix (0.2% Se); 0.083% iron sulfate; 0.010% vitamin A (1,000,000 IU/g; DSM Nutritional Products, Inc., Parsippany, NJ); 0.003% ethylenediamine dihydroiodide; and 0.002% cobalt carbonate.

^5^As measured by proximate analysis of bunk samples.

### Experimental Design, Treatments, and Pen Assignment

A completely randomized block design was used. Steers were weighed 7 and 3 d prior to initiation of the experiment. An average of the two body weights were used to stratify steers into four weight blocks with four pens in each block and two steers per pen. Steers were housed in concrete, partially slatted floor pens (5.5 × 2.9 m, 2.4 m of linear bunk space) and had ad libitum access to water. Within each block, pens were randomly assigned to treatment: (1) 0 mg Cr/kg to diet DM (control), (2) 0.15 mg Cr/kg to diet DM, (3) 0.30 mg Cr/kg to diet DM, and (4) 0.45 mg Cr/kg to diet DM. Treatments were formulated using CrP (KemTRACE Chromium, Kemin Industries, Inc.) with ground corn as a carrier with separate top-dress mixtures for each specific treatment. The control treatment top-dress consisted of 100% ground corn. Treatments were administered to pens by top-dressing the ration daily immediately following feed delivery and thoroughly mixed into each pen’s feed using a treatment specific hand-held scoop. Orts were collected if carryover feed went out of condition or was present on weigh days, dried for 24 h at 100 °C, and deducted from the intake file in order to calculate dry matter intake (DMI). Bunk samples were obtained for each interim period and proximate analysis was performed using AOAC procedures at a commercial feed analysis laboratory (Servi-Tech Laboratories, Amarillo, TX; Table 1).

### Sample and Data Collection

Body weights and blood samples were collected on days 0, 28, 56, 91, 119, and 147. Blood samples were obtained using jugular venipuncture using silicone coated glass vacuum blood collection tubes (Vacutainer, BD Diagnostics, Franklin Lakes, NJ). Tubes were stored at 4 °C overnight to allow for clotting, then centrifuged for 20 min at 1,500 × *g* to harvest sera. Sera samples were then stored at −20 °C until subsequent laboratory analyses. Sera samples were analyzed for non-esterified fatty acid (NEFA) content using the NEFA-HR(2) kit (Wako Diagnostics, Richmond, VA), glucose using an enzymatic Autokit Glucose kit (Wako Diagnostics), insulin using a bovine-specific insulin ELISA (Alpco Diagnostics, Salem, NH), and serum urea-N (SUN) using a commercially available kit (Nitrogen Liqui-UV kit; Stanbio Laboratory, Boerne, TX).

Steers were transported 288 km to a commercial abattoir for harvest on day 150. Carcass data were collected by West Texas A&M University Beef Carcass Research Center personnel and included hot carcass weight (HCW), subcutaneous fat thickness opposite the longissimus muscle at the 12th rib (RF), longissimus muscle area at the 12th rib, kidney-pelvic-heart fat percentage of hot carcass weight, calculated yield grade (YG), and marbling score (small^00^ = 400). Dressing percentage (DP) was calculated as hot carcass weight divided by body weight (BW) that was pencil shrunk 4% on day 150. Carcass ADG was calculated assuming an initial DP of 58% on day 0, as suggested by Rathmann et al. (2012). Equations described by Guiroy et al. (2002) were used to calculate predicted empty body fat percentage (EBF), empty body weight (EBW), and final BW when EBF was adjusted to 28% EBF.

### Biopsy Sample Collection

To evaluate biochemical changes in skeletal muscle due to CrP supplementation, biopsy samples were obtained from the longissimus muscle on days 0, 28, 56, 91, 119, and 147, prior to feeding. To collect the skeletal muscle biopsy sample, steers were restrained in a hydraulic squeeze chute and the area surrounding the incision site was shaved using a disposable razor (Bic, Milford, CT). The area was then scrubbed with sterile gauze using water, a 7.5% povidone iodine surgical scrub (Betadine, Purdue Products, L.P., Stamford, CT), and 70% ethanol. A local anesthetic (lidocaine HCl, 20 mg/mL) was injected into the subcutaneous fat layer (8 mL per biopsy) in a 6 cm^2^ square-shaped pattern (4 injection sites, 2 mL lidocaine HCl per site), and a minimum of 5 min was allowed for the local anesthetic to induce numbness. Once the area was numb, 70% ethanol and sterile gauze was again used to have aseptic preparation of the surgical area; then a 1-cm long incision was made using a sterile scalpel. A 6-mm diameter Bergstrom biopsy needle was used to collect ~2 g of longissimus tissue. The sample was placed on sterile gauze in a covered plastic container to be taken to the sample preparation area. The incision was closed using veterinary tissue adhesive (VetBond, 3M Animal Care Products, St. Paul, MN) and sprayed with a protective spray (AluShield, Neogen Corp, Lexington, KY) to aid in preventing infection during the healing process. Biopsies alternated sides of the animal and the incision site moved anterior 2 cm to the previous incision on that side.

Once the sample was taken to the sample preparation area, the ~2 g tissue was divided into three portions. One portion (~0.25 g) to be used for immunohistochemical (IHC) analysis was analyzed under a magnifying glass, and muscle fibers were identified. The fibers were placed parallel to each other on a 1 cm × 1.5 cm piece of corkboard and frozen in clear frozen section compound (VWR International, West Chester, PA) using isopentane cooled with dry ice. The samples on cork pieces were wrapped in foil, placed in sample bags (Whirl-Pak, NASCO, Fort Atkinson, WI), and stored on dry ice until transport back to Texas Tech University. The other two portions, one for RNA analysis and the other for protein analysis, were placed in sample bags (Whirl-Pak), frozen in liquid nitrogen, and stored on dry ice until transport back to Texas Tech University. All skeletal muscle samples were stored at −80 °C until further processing.

### RNA Isolation and RT-qPCR

To isolate RNA, ~0.5 g of each sample was homogenized with 3 mL of TRI Reagent (Sigma, St. Louis, MO), then chloroform was added. The mixture was centrifuged, and the upper aqueous layer was transferred to a new microcentrifuge tube. To precipitate the RNA from solution, ice-cold isopropanol was added. After centrifugation, the supernatant was removed. The RNA pellet was allowed to air dry, and then 70% ethanol was added to the tube. To quantify RNA concentration, the supernatant was again removed, and the pellet was allowed to air dry. The pellet was dissolved in nuclease-free water, and RNA concentration and purity (260/280 ratio) was measured using a NanoDrop 1000 spectrophotometer (NanoDrop Products, ThermoFisher Scientific, Waltham, MA). Synthesis of cDNA and removal of possible genomic DNA contamination was performed using the QuantiTect Reverse Transcription kit (Qiagen, Germantown, MD.) according to the manufacturer’s recommendations. The expression of AMP-activated protein kinase α (AMPKα), GLUT4, insulin-like growth factor I (IGF-I), myosin heavy chain isoform I (MHC-I), myosin heavy chain isoform IIA (MHC-IIA), myosin heavy chain isoform IIX (MHC-IIX), and peroxisome proliferator-activated receptor γ (PPARγ; Table 2) were determined using real-time quantitative PCR on a GeneAmp 7900HT Sequence Detection System (Applied Biosystems, ThermoFisher) with ribosomal protein subunit 9 as the housekeeping gene. Cycle length, temperature, and repetition were run according to the manufacturer’s recommendation for TaqMan Master Mix polymerase (ThermoFisher). Relative expression was calculated using a composite sample as an internal reference standard.

**Table 2.** Sequence for bovine PCR primers and TaqMan probes for genes of interest

Item	Sequence (5′ to 3′)
*AMPK-α (accession no. NM_001109802)*	
Forward	ACCATTCTTGGTTGCTGAAACTC
Reverse	CACCTTGGTGTTTGGATTTCTG
TaqMan probe	6FAM-CAGGGCGCGCCATACCCTTG-TAMRA
*GLUT4 (accession no. D63150)*	
Forward	CCTCGGCAGCGAGTCACT
Reverse	AAACTGCAGGGAGCCAAGAA
TaqMan probe	CCTTGGTCCTTGGCGTATTCTCCGC
*IGF-I (accession no. X15726)*	
Forward	TGTGATTTCTTGAAGCAGGTGAA
Reverse	AGCACAGGGCCAGATAGAAGAG
TaqMan probe	6FAM-TGCCCATCACATCCTCCTCGCA-TAMRA
*MHC-I (accession no. AB059400)*	
Forward	CCCACTTCTCCCTGATCCACTAC
Reverse	TTGAGCGGGTCTTTGTTTTTCT
TaqMan probe	6FAM-CCGGCACGGTGGACTACAACATCATAG-TAMRA
*MHC-IIA (accession no. AB059398)*	
Forward	GCAATGTGGAAACGATCTCTAAAGC
Reverse	GCTGCTGCTCCTCCTCCTG
TaqMan probe	6FAM-TCTGGAGGACCAAGTGAACGAGCTGA-TAMRA
*MHC-IIX (accession no. AB059399)*	
Forward	GGCCCACTTCTCCCTCATTC
Reverse	CCGACCACCGTCTCATTCA
TaqMan probe	6FAM-CGGGCACTGTGGACTACAACATTACT-TAMRA
*PPARγ (accession no. NM_181024)*	
Forward	ATCTGCTGCAAGCCTTGGA
Reverse	TGGAGCAGCTTGGCAAAGA
TaqMan probe	6FAM-CTGAACCACCCCGAGTCCTCCCAG-TAMRA
*RPS9 (accession no. DT860044)*	
Forward	GAGCTGGGTTTGTCGCAAAA
Reverse	GGTCGAGGCGGGACTTCT
TaqMan probe	6FAM-ATGTGACCCCGCGGAGACCCTTC-TAMRA

^1^AMPKα = AMP-activated protein kinase α, GLUT4 = glucose transporter 4, IGF-I = insulin-like growth factor I, MHC-I = myosin heavy chain-I, MHC-IIA = myosin heavy chain-IIA, MHC-IIX = myosin heavy chain-IIX, and RPS9 = ribosomal protein subunit 9.

### Protein Isolation and Western Blotting

Samples to be used for protein expression analysis were homogenized with whole muscle extraction buffer (WMEB; 2%, w/v, sodium dodecyl sulfate, 10 mM phosphate, pH 7.0). For each gram of biopsied LM tissue, 5 mL of WMEB was used. After centrifugation of the homogenate, the middle aqueous layer was extracted and placed in microcentrifuge tubes. The concentration of total protein was determined with a Pierce BCA protein assay (ThermoFisher) and spectrophotometry (NanoDrop 1000, ThermoFisher). Samples were then diluted to a constant concentration using WMEB and added to a modified Wang’s Tracking Dye. To create reducing conditions, β-mercaptoethanol was added. Five micrograms of total protein per sample were loaded into precast Novex 4% to 12% Bis-Tris gels (Invitrogen, ThermoFisher), and run in MES buffer (ThermoFisher) according to manufacturer directions. An iBlot transfer device was used to transfer proteins from the gel to a nitrocellulose membrane (ThermoFisher). Nonspecific antibody binding was inhibited by incubating membranes with 5% nonfat dry milk (Bio-Rad, Hercules, CA) in 1× Tris-buffered saline (TBS) at room temperature for 1 h. Diluted primary antibody in 1× TBS-0.04% Tween (1:1,000 α-GLUT4, rabbit IgG; Abcam, Cambridge, MA) was added to each membrane and allowed to incubate while gently rocking overnight at 4 °C. Membranes were rinsed with 1× TBS-0.04% Tween, then incubated in the dark with a fluorescent secondary antibody (1:2,000 Alexa Fluor 633, goat α-rabbit IgG; ThermoFisher) for 1 h at room temperature. After rinsing with 1× TBS-0.04% Tween, membranes were dried, preventing light exposure, then imaged with a VersaDoc Image Scanner II and ImageQuant TL software. Densitometry values for each sample were normalized to an internal reference standard present on each membrane and reported as a ratio. Bands corresponding to the target protein were identified using a molecular weight standard present on each gel (Precision Plus Protein, All Blue Standards; Bio-Rad).

### Immunohistochemical Analysis

Embedded samples for IHC staining were used to determine muscle fiber distribution, cross-sectional area, GLUT4 density, and satellite cell populations were moved from −80 to −20 °C to thaw for 24 h to prevent shattering during the cryostat sectioning procedure. Samples were removed from the cork and cut into 10 µm-thick cross-sections at −20 °C with a Leica CM1950 cryostat (Leica Biosystems, Buffalo Grove, IL) and placed on positively charged glass slides (four slides per sample, five cross-sections per slide; Superfrost Plus, VWR International). Cross-sections were fixed with 4% paraformaldehyde in phosphate-buffered saline (PBS) (ThermoFisher) for 10 min at room temperature, then rinsed in PBS at room temperature. A blocking solution consisting of 2% bovine serum albumin (MD Biomedical, Solon, OH), 5% horse serum (Invitrogen), and 0.2% Triton X-100 (ThermoFisher) in PBS was then applied to the fixed cross-sections and allowed to incubate for 30 min at room temperature to prevent nonspecific antibody binding. The cross-sections were incubated with primary antibodies in blocking solution for 1 h at room temperature. The following primary antibodies were used: slide 1: 1:100 α-dystrophin, rabbit, IgG (ThermoFisher); 1:100 supernatant anti-MHC type 1, IgG2b (BA-D5; Developmental Studies Hybridoma Bank, University of Iowa, Iowa City, IA); and supernatant anti-MHC all but type IIX IgG1 (BF-35, Developmental Studies Hybridoma Bank); slide 2: 1:500 GLUT4 (Abcam); and slide 3: 1:10 anti-PAX7 (paired box protein 7) supernatant (mouse α-chicken; Developmental Studies Hybridoma Bank), and 1:100 Myf-5 (myogenic factor 5; rabbit IgG; Santa Cruz Biotechnology, Dallas, TX). The cross-sections were rinsed in PBS at room temperature. The following secondary antibodies in blocking solution were then applied to the cryosections for 30 min at room temperature in the dark: slide 1: 1:1,000 goat α-rabbit, IgG, Alexa Fluor 488 (ThermoFisher), 1:1,000 goat α-mouse, IgG1, Alexa Fluor 546 (ThermoFisher), 1:1,000 goat α-mouse, IgG2b, Alexa Fluor 633 (ThermoFisher); slide 2: 1:1,000 rabbit α-mouse, IgG2a, Alexa Fluor 546 (ThermoFisher); and slide 3: 1:1,000 Alexa Fluor 488 (goat α-rabbit IgG; ThermoFisher), and 1:1,000 Alexa Fluor 546 (goat α-mouse IgG1; ThermoFisher). Following the incubation period, the slides were rinsed three times with PBS for 5 min at room temperature. The slides used for muscle fiber type and area were then cover-slipped using thin glass coverslips (VWR International) and ProLong Gold with 4′6-diamidino-2-phenylindole mounting media (ThermoFisher) and allowed to cure in the dark for 36 h at room temperature. Slides used for GLUT4 density and satellite cell populations were cover-slipped with AquaMount mounting media (Lerner Laboratories, Pittsburgh, PA) and thin glass coverslips (VWR International) and cured for 24 h at 4 °C in the dark. The slides were imaged at 200× working magnification with an inverted fluorescence microscope (Nikon Eclipse, Ti-E; Nikon Instruments Inc., Mellville, NY) using a UV light source (Nikon Intensilight; C-HGFIE), and images were taken using a CoolSnap ES2 monochrome camera. The images were then artificially colored and analyzed with NIS-Elements AR software.

### Statistical Analysis

All statistical analysis was performed using SAS version 9.4 (SAS Institute, Inc., Cary, NC). For steer growth performance data, pen was an experimental unit, block was a random effect, and treatment was included as the fixed effect. For sera parameters, gene expression, protein expression, and IHC analyses, animal served as the experimental unit; the random effect of block and fixed effect of treatment were used. Due to poor feed intake and performance, one pen was removed from live and carcass data analysis. For gene expression analysis, samples were run in triplicate and averaged. For IHC staining, five images were taken of each sample. For fiber type distribution, satellite cell density, and GLUT4 density, the measurements from the five images per sample were averaged. For the fiber cross-sectional area, the area of each fiber type per sample was averaged. The average of each fiber type for each sample was then subjected to statistical analysis. Day was used as a repeated measure for sera, gene expression, protein expression, and IHC data, and the covariance structure with the best fit was utilized as determined using the lowest Akaike information criterion. The GLIMMIX procedure was used with the Kenward–Roger adjustment to correct the degrees of freedom and the least squares means procedure and pairwise comparisons PDIFF option was used to separate treatment means. Means were also separated using orthogonal linear and quadratic contrasts (Steel and Torrie, 1960). Means were determined to be significantly different when *P* ≤ 0.05. When 0.05 > *P* ≤ 0.10, a tendency was declared.

## RESULTS AND DISCUSSION

### Live Performance

Supplementation of Cr improved BW and ADG. The 0.45 mg of Cr/kg of DM treatment was heavier (*P* < 0.05) starting on day 56 and remained the heaviest treatment group throughout the remainder of the experiment (Table 3). There was also a linear treatment effect (*P* ≤ 0.01) for BW on day 56 until day 147. For the first 56 d of the experiment, there was a linear treatment relationship (*P* < 0.05) for ADG with the 0.45 mg/kg treatment having the greatest ADG. This pattern was also present for the first 119 d (*P* < 0.01) and the duration of the trial (*P* = 0.03). However, there was no difference in ADG for days 119 to 147 (*P* > 0.10) during ractopamine HCl supplementation. There was no effect on DMI (*P* > 0.10) throughout the entire feeding period, but feed efficiency was linearly improved for the entire feeding period with the 0.45 mg/kg treatment having the most efficient G:F (*P* = 0.01). The observation of improved BW is similar to results reported in Mexico, where bulls fed Cr-methionine were heavier than the control group for animals fed in both cool and hot, humid seasons (Barajas et al., 2008a, 2008b). Additionally, the present study supports previous data where supplementation of CrP during the receiving period of feedlot steers resulted in a positive linear response for ADG (Bernhard et al., 2012). Herkelman et al. (2018) also showed a tendency for Cr supplementation to improve ADG of steers during the receiving period. Supplementation of high Cr-yeast also improved ADG in stressed feeder calves (Moonsie-Shangeer and Mowat, 1993). The lack of difference between treatments during the ractopamine HCl period is similar to several studies where no difference was observed in cattle fed ractopamine HCl only or a combination of CrP and ractopamine HCl (Bohrer et al., 2014; Edenburn et al., 2016). This is interesting, as the modes of action for CrP and ractopamine HCl alter responses through differing pathways. Cr augments the insulin response through the protein chromodulin, which activates the tyrosine kinases of insulin receptors to improve clearance of circulating glucose from the blood into insulin sensitive tissues (Davis and Vincent, 1997). Moonsie-Shangeer and Mowat (1993) found that supplementation of high Cr-yeast increased DMI, which is in contrast to the present study. However, the steers in the present study were heavier at trial initiation and were not subjected to the same distance of transportation prior to arrival at the feedlot. The lack of stress could explain the contrasting results of DMI.

**Table 3.** Effects of Cr propionate supplementation on live performance of feedlot steers^#^

	Treatment, mg Cr/kg^1^						Contrasts	
Item	0.00	0.15	0.30	0.45	SEM^2^	*P*-value	Linear	Quadratic
Weight, kg								
Day0	366.1	365.7	368.1	368.0	2.48	0.65	0.27	0.94
Day28	418.6	416.2	426.2	430.4	9.18	0.31	0.10	0.64
Day56	464.6^b^	464.0^b^	481.4^ab^	493.6^a^	11.45	0.03	<0.01	0.42
Day91	535.1^b^	521.8^b^	541.5^ab^	562.8^a^	11.80	0.01	0.01	0.04
Day119	574.8^bc^	559.1^c^	590.8^ab^	615.4^a^	15.27	<0.01	<0.01	0.06
Day147	602.3^b^	594.5^b^	615.3^ab^	638.0^a^	15.18	0.02	0.01	0.15
ADG, kg								
Days0–56	1.76^b^	1.76^b^	2.00^ab^	2.24^a^	0.192	0.04	0.01	0.37
Days56–119	1.75	1.51	1.74	1.94	0.176	0.10	0.14	0.08
Days0–119	1.76^b^	1.63^b^	1.87^ab^	2.08^a^	0.122	<0.01	<0.01	0.05
Days119–147	0.98	1.27	0.94	0.81	0.288	0.40	0.33	0.30
Days0–147	1.61^b^	1.56^b^	1.69^ab^	1.84^a^	0.098	0.03	0.01	0.15
DMI, kg								
Days0–56	8.1	7.9	8.4	8.3	0.28	0.35	0.20	0.86
Days56–119	9.1	8.8	9.1	9.8	0.47	0.26	0.15	0.18
Days0–119	8.6	8.4	8.8	9.1	0.36	0.30	0.14	0.31
Days119–147	8.6	8.5	8.6	9.3	0.47	0.33	0.16	0.27
Days0–147	8.6	8.4	8.8	9.1	0.34	0.23	0.11	0.25
G:F								
Days0–56	0.219	0.220	0.228	0.269	0.0203	0.09	0.02	0.32
Days56–119	0.193	0.170	0.194	0.200	0.0203	0.47	0.50	0.34
Days0–119	0.204^b^	0.193^b^	0.214^ab^	0.229^a^	0.0095	0.02	0.01	0.07
Days119–147	0.114	0.151	0.111	0.087	0.0394	0.43	0.33	0.29
Days0–147	0.196^b^	0.193^b^	0.201^ab^	0.211^a^	0.0052	0.03	0.01	0.11

^ab^Means within same row with different superscripts differ (*P* < 0.05).

^#^ *n* = 4.

^1^Treatments were formulated to consist of 0.00 mg Cr/kg DM, 0.15 mg Cr/kg diet DM, 0.30 mg Cr/kg diet DM, and 0.45 mg Cr/kg diet DM (Cr propionate; KemTRACE Chromium, Kemin Industries, Inc., Des Moines, IA).

^2^Pooled standard error of the mean.

### Carcass Trait Responses

As shown in Table 4, the pattern presented for live weight continued with a positive linear response for HCW (*P* < 0.01). Carcasses from cattle supplemented with 0.45 mg/kg Cr were 28-kg heavier than control carcasses. There was a numerically linear increase in marbling scores; however, no effect of treatment on marbling score was identified (*P* > 0.10). There was no difference (*P* > 0.10) for any other carcass trait characteristics measured in the present study. This is comparable to previous data where supplementation of Cr resulted in heavier carcass weight (Kneeskern et al., 2016; Rode et al., 2016). However, the level of Cr supplementation was 0.30 mg/kg in previous studies, compared with increments up to 0.45 mg/kg used in the present study, and this is in agreement with several studies (Barajas et al., 2008a, 2008b; Bohrer et al., 2014; Edenburn et al., 2016). The action of Cr supplementation increasing glucose uptake by cells could allow for increased marbling, as intramuscular adipocytes which constitute marbling have been shown to prefer glucose as a substrate for lipid accumulation (Smith and Crouse, 1984); however, no differences in marbling were detected in the present study.

**Table 4.** Effects of Cr supplementation on carcass characteristics of feedlot steers^#^

	Treatment, mg Cr/kg^1^						Contrasts	
Item	0.00	0.15	0.30	0.45	SEM^2^	*P*-value	Linear	Quadratic
Hot carcass weight, kg	394.5^b^	395.0^b^	404.4^ab^	422.8^a^	10.54	0.02	<0.01	0.22
DP	62.89	63.87	63.08	63.46	1.431	0.89	0.83	0.76
Marbling score^3^	485.0	502.5	520.4	521.3	48.3	0.83	0.38	0.80
12th rib fat thickness, cm	1.47	1.30	1.12	1.42	0.361	0.38	0.73	0.31
Ribeye area, cm^2^	87.7	92.3	91.0	92.9	4.58	0.79	0.44	0.81
KPH, %	2.1	2.2	2.1	2.1	0.21	0.94	0.70	0.64
Calculated YG	2.73	2.95	2.71	3.24	0.487	0.64	0.38	0.65

^ab^Means within same row with different superscripts differ (*P* < 0.05).

^#^ *n* = 4.

^1^Treatments were formulated to consist of 0.00 mg Cr/kg DM, 0.15 mg Cr/kg diet DM, 0.30 mg Cr/kg diet DM, and 0.45 mg Cr/kg diet DM (Cr propionate (KemTRACE Chromium, Kemin Industries, Inc., Des Moines, IA).

^2^Pooled standard error of the mean.

^3^400 = Small^00^, 500 = Modest^00^.

### Estimated Carcass Performance

When ADG is compared on a carcass basis, the impact of CrP supplementation was similar to what was seen for live-basis ADG. A linear effect was seen (*P* = 0.01; Table 5) with carcass ADG increasing as CrP in the diet increased. However, when G:F is evaluated on a carcass gain basis, a tendency (*P* = 0.09) for a linear effect of treatment was observed. When evaluating the ratio of carcass ADG to live ADG, no difference (*P* > 0.10) between treatment groups was detected. No difference (*P* > 0.10) between treatments was shown for percent EBF. Similar to live BW and HCW, a linear effect (*P* = 0.01) was observed for the effect of CrP on EBW, with increased values with increased CrP supplementation. When final shrunk BW was adjusted to a constant 28% EBF, no difference (*P* > 0.10) existed between treatment groups. Furthermore, the ratio of carcass ADG:live ADG is increased, meaning more weight gain is directed toward carcass tissues than the whole body. The present study did not see this response due to CrP supplementation, meaning the observed increases in BW and HCW were due to whole body growth rather than specifically growth of carcass tissues.

**Table 5.** Effects of Cr supplementation on estimated carcass characteristics on a live basis of feedlot steers^#1^

	Treatment, mg Cr/kg^2^						Contrasts	
Item	0.00	0.15	0.30	0.45	SEM^3^	*P*-value	Linear	Quadratic
Carcass ADG, kg	1.21^b^	1.22^b^	1.27^ab^	1.40^a^	0.069	0.03	0.01	0.23
Carcass G:F	0.148	0.151	0.151	0.160	0.0070	0.30	0.09	0.56
Carcass ADG:live ADG, %	70.0	72.4	70.0	70.4	3.43	0.87	0.90	0.67
EBF,^4^ %	30.84	29.79	29.26	30.92	1.792	0.69	0.96	0.25
EBW,^5^ kg	551.4^b^	552.1^b^	564.5^ab^	588.7^a^	13.87	0.02	0.01	0.22
28% EBF adjusted final BW,^6^ kg	504.8	523.5	534.3	541.9	34.67	0.68	0.23	0.80

^ab^Means within same row with different superscripts differ (*P* < 0.05).

^#^ *n* = 4

^1^Carcass ADG, carcass G:F, and carcass ADG:live ADG were calculated assuming a 58% DP on day 0.

^2^Treatments were formulated to consist of 0.00 mg Cr/kg DM, 0.15 mg Cr/kg diet DM, 0.30 mg Cr/kg diet DM, and 0.45 mg Cr/kg diet DM (Cr propionate (KemTRACE Chromium, Kemin Industries, Inc., Des Moines, IA).

^3^Pooled standard error of the mean.

^4^EBF (%) = 17.76207 + (4.68142 × 12th-rib fat thickness) + (0.01945 × HCW) + (0.81855 × quality grade) – (0.06754 × ribeye area). Numerical quality grade was assigned where low choice = 5 to 6 and average choice = 6 to 7; Guiroy et al. (2002).

^5^EBW = (1.316 × HCW) + 32.29; Guiroy et al. (2002).

^6^Final shrunk body weight adjusted to 28% EBF = EBW = [(28 – EBF) × 14.26]/0.891; Guiroy et al. (2002).

### Sera Metabolite Parameters

There was no treatment effect nor interaction of treatment and day (*P* > 0.05) on any sera parameters measured. However, a day effect (*P* < 0.05) was observed for insulin, SUN, and NEFA concentrations, while day tended to impact sera glucose levels (*P* < 0.10), as shown in Table 6. This is similar to previous research showing no difference in sera metabolites in receiving steers after 56 d of CrP supplementation (Bernhard et al., 2012). Additionally, in native Korean cattle, supplementation of Cr-methionine to low forage diets did not affect circulating concentrations of urea nitrogen, insulin, or glucose (Sung et al., 2015). However, these cattle were not subjected to glucose or insulin tolerance tests and neither were steers in the present study. The observed results differ from previous research where Cr supplementation improved glucose clearance by decreasing the levels of circulating glucose (Kegley et al., 2000; Spears et al., 2012). In steers undergoing a glucose tolerance test, supplementation of CrP did not impact insulin or glucose levels (Kneeskern et al., 2016). It should be noted that glucose or insulin tolerance tests were not administered to cattle in the present study.

**Table 6.** Effects of Cr supplementation on sera metabolites of feedlot steers^#^

	Day							Treatment, mg/kg Cr^1^					*P*-values^#^		
Item^*^	0	28	56	91	119	147	SEM	0	0.15	0.30	0.45	SEM^2^	*D*	*T*	*D* × *T*
Glucose	104.6	97.9	94.4	101.9	90.1	86.4	7.48	100.5	93.1	90.4	99.5	6.38	0.07	0.26	0.46
Insulin	1.99^wx^	2.44^xy^	2.70^y^	3.41^z^	2.98^yz^	1.44^w^	0.700	2.37	2.86	1.98	2.76	0.625	<0.01	0.50	0.76
SUN	11.9^x^	11.9^x^	17.1^z^	14.4^y^	13.8^y^	15.0^y^	0.86	13.7	14.3	14.5	13.7	1.07	<0.01	0.82	0.21
NEFA	0.29^xy^	0.24^x^	0.26^xy^	0.30^y^	0.37^z^	0.27^xy^	0.030	0.31	0.27	0.29	0.29	0.038	<0.01	0.75	0.94

^wxyz^Means within same row with different superscripts differ for effect of day (*P* < 0.05).

^#^ *n* = 4.

*Glucose: mg/dL; insulin: ng/mL; SUN: serum urea nitrogen, mg/dL; NEFA: non-esterified fatty acids, mmol/L.

^#^ *D*,- day; *T*, treatment; *D* × *T*, day by treatment interaction.

^1^Treatments were formulated to consist of 0.00 mg Cr/kg DM, 0.15 mg Cr/kg diet DM, 0.30 mg Cr/kg diet DM, and 0.45 mg Cr/kg diet DM (Cr propionate (KemTRACE Chromium, Kemin Industries, Inc., Des Moines, IA).

^2^Pooled standard error of the mean.

### Gene and Protein Expression

No interaction of day and CrP treatment was observed; therefore, only main effects are discussed (Table 7). No difference in expression of AMPKα was detected across treatments (*P* > 0.05; Table 7), but a tendency for a day effect was seen (*P* = 0.054). Differences in the expression of IGF-I, MHC-I, MHC-IIA, MHC-IIX, and PPARγ were seen over time (*P* < 0.05). The only gene for which expression was changed due to treatment was PPARγ (*P* = 0.05), where expression was decreased in the 0.15 and 0.30 mg/kg CrP treatments. This is in contrast to others, where PPARγ mRNA expression was not changed due to addition of CrP to the cell culture media of bovine satellite cells (Tokach et al., 2015). In bovine intramuscular adipocytes, adding Cr-acetate to the cell culture media altered expression of AMPKα (Kim et al., 2020). Additionally, Tokach et al. (2015) reported no change in expression of AMPKα, MHC-I, MHC-IIA, or MHC-IIX, in agreement with the present study.

**Table 7.** Effects of Cr supplementation on relative gene expression in longissimus biopsy samples of feedlot steers^#^

	Day							Treatment, mg/kg Cr					*P*-values^#^		
Gene^*^	0	28	56	91	119	147	SEM^2^	0	0.15	0.30	0.45	SEM	*D*	*T*	*D* × *T*
AMPKα	2.75	2.70	1.90	1.93	2.31	3.28	0.771	3.11	2.64	2.32	1.83	0.575	0.06	0.14	0.74
IGF-I	1.69^xy^	1.62^xy^	1.74^xy^	2.87^z^	2.35^yz^	1.47^x^	0.415	1.98	1.86	1.60	2.38	0.393	<0.01	0.26	0.85
PPARγ	1.90^y^	4.47^z^	2.99^yz^	2.77^yz^	3.53^z^	3.77^z^	1.001	3.84^a^	3.13^b^	1.98^b^	4.02^a^	0.833	0.02	0.05	0.32
MHC-I	2.17^y^	0.92^x^	2.00^y^	2.55^y^	3.45^z^	4.02^z^	0.730	2.57	2.81	2.50	2.19	0.433	<0.01	0.53	0.18
MHC-IIA	2.19^yz^	0.86^x^	1.39^xy^	2.42^z^	3.08^z^	3.12^z^	0.832	1.94	2.68	2.19	1.89	0.510	<0.01	0.35	0.80
MHC-IIX	3.46^z^	1.26^x^	1.59^x^	2.21^y^	3.18^z^	4.14^z^	0.816	2.48	2.85	2.90	2.33	0.511	<0.01	0.55	0.85

^xyz^Means within same row with different superscripts differ for effect of day (*P* < 0.05).

^ab^Means within same row with different superscripts differ for effect of treatment (*P* < 0.05).

^#^ *n* = 4.

*AMPKα = AMP-activated protein kinase alpha; IGF-I = insulin-like growth factor I; MHC-I = myosin heavy chain-I; MHC-IIA = myosin heavy chain-IIA; and MHC-IIX = myosin heavy chain-IIX. RPS9 used as housekeeper gene.

^#^ *D*, day; *T*, treatment; *D* × *T*, day by treatment interaction.

^2^Pooled standard error of the mean.

**Figure 1.**
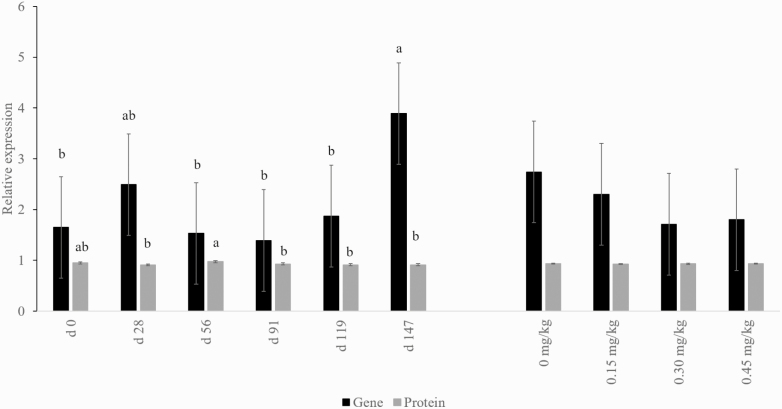
Effects of CrP supplementation on relative gene and protein expression of GLUT-4 in longissimus biopsies of feedlot steers collected on days 0, 28, 56, 91, 119, and 147 of the feeding trial (*n* = 4). Treatments were 0 mg Cr/kg diet DM, 0.15 mg Cr/kg diet DM (CrP; KemTRACE Chromium 0.04%, Kemin Industries, Des Moines, IA), 0.30 mg Cr/kg diet DM, and 0.45 mg Cr/kg diet DM. Differing superscripts indicate expression differs for day (gene *P*= 0.04; protein *P* < 0.01).

As shown in Figure 1, gene and protein expression of GLUT4 was impacted by day (*P* < 0.001), but not altered by supplementation of CrP (*P* > 0.05). This is similar to findings by Tokach et al. (2015), where skeletal muscle satellite cells were incubated with media containing various concentrations of CrP with no differences in mRNA expression for GLUT4. In the same study, addition of CrP decreased protein expression of GLUT4. However, when rat L6 myoblasts were treated with Cr and insulin, there was an increase in GLUT4 mRNA expression compared with controls (Qiao et al., 2009). These prior findings were observed in vitro, while the in vivo data of the present study where animals were supplemented with Cr with no impact on total expression of GLUT4.

### Immunohistochemical Analysis

**Figure 2.**
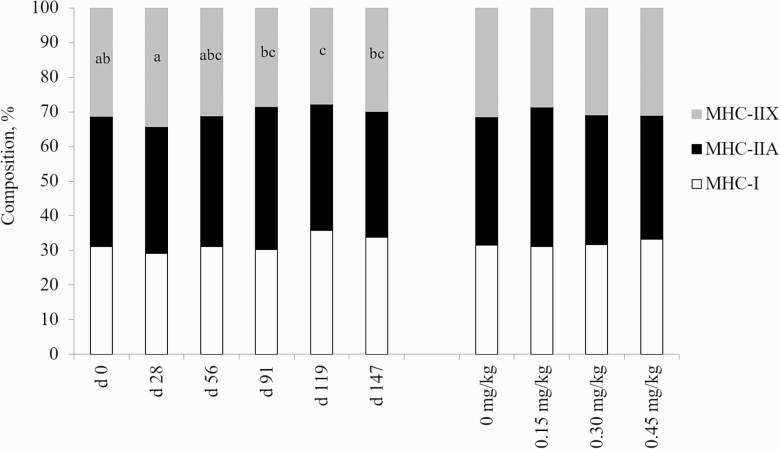
Effects of CrP supplementation on skeletal muscle fiber type distribution in feedlot steers from longissimus biopsies collected on days 0, 28, 56, 91, 119, and 147 of the feeding trial (*n* = 4). Treatments were 0 mg Cr/kg diet DM, 0.15 mg Cr/kg diet DM (CrP; KemTRACE Chromium 0.04%, Kemin Industries, Des Moines, IA), 0.30 mg Cr/kg diet DM, and 0.45 mg Cr/kg diet DM. Cross-sections of skeletal muscle samples were stained by immunohistochemistry for presence of myosin heavy chain (MHC) isoforms. Differing superscripts denote means within MHC isoform IIX differ by day (*P* = 0.01).

For the fiber type distribution, there were no interactions of treatment and day present (*P* < 0.05). As displayed in Figure 2, the proportion of MHC-I and MHC-IIA fibers tended to increase from days 0 to 147 (*P* = 0.062 and 0.075, respectively). A day effect was observed for MHC-IIX fibers, with the proportion of MHC-IIX fibers decreasing until day 119, then increasing on day 147, which was not different from day 0. This reflects the mRNA expression data of no change in expression of any of the three skeletal muscle fiber types found in cattle. This is congruent with established data where fiber type proportions were not different in animals harvested after varying days on feed (Johnston et al., 1975).

**Figure 3.**
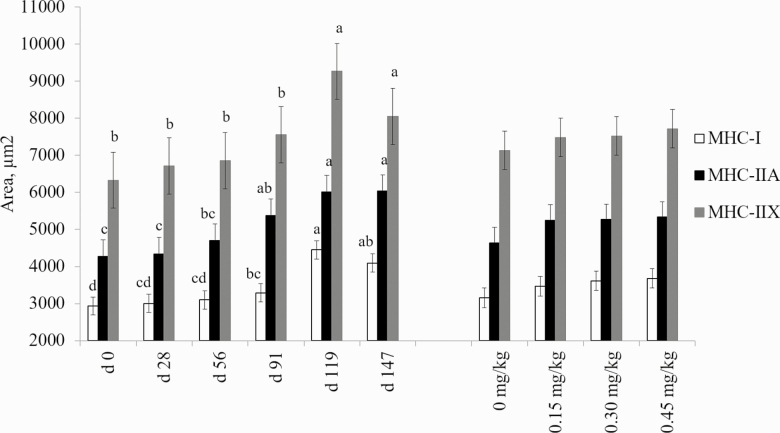
Effects of CrP supplementation on skeletal muscle fiber area in feedlot steers from longissimus biopsies collected on days 0, 28, 56, 91, 119, and 147 of the feeding trial (*n* = 4). Treatments were 0 mg Cr/kg diet DM, 0.15 mg Cr/kg diet DM (CrP; KemTRACE Chromium 0.04%, Kemin Industries, Des Moines, IA), 0.30 mg Cr/kg diet DM, and 0.45 mg Cr/kg diet DM. Cross-sections of skeletal muscle samples were stained by immunohistochemistry for presence of myosin heavy chain (MHC) isoforms. Differing superscripts denote means within MHC isoform differ by day (*P* < 0.01).

**Figure 4.**
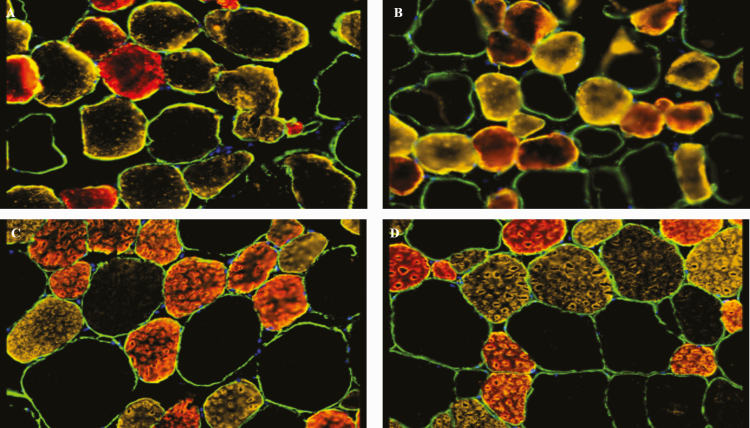
Immunohistochemical staining of skeletal muscle fibers in feedlot steers from longissimus biopsies (*n* = 4). Panel A: day 0, 0 mg Cr/kg; (panel B) day 147, 0 mg Cr/kg diet DM; (panel C) day 0, 0.45 mg Cr/kg (CrP; KemTRACE Chromium 0.04%, Kemin Industries, Des Moines, IA); (panel D) day 147, 0.45 mg Cr/kg. Green-sarcolemma, red-myosin heavy chain (MHC)-I fibers, yellow-MHC-IIA fibers, black- MHC-IIX fibers, blue- nuclei.

No interaction between treatment and day was observed for cross-sectional area for any fiber types measured in the present study. Supplementation with CrP did not change cross-sectional area for any fiber type in bovine skeletal muscle (*P* > 0.05; Figure 3). However, as days on feed increased, the cross-sectional area of all three fiber types increased (*P* < 0.001). This was expected, as it has been shown that skeletal muscle fiber cross-sectional area in the longissimus dorsi increases as animals advance in age (Joubert, 1956; Tuma et al., 1962; Johnston et al., 1975; Figure 4). Hypertrophy of skeletal muscle fibers is the cause of skeletal muscle growth postnatal, as the number of skeletal muscle fibers is fixed after birth.

**Figure 5.**
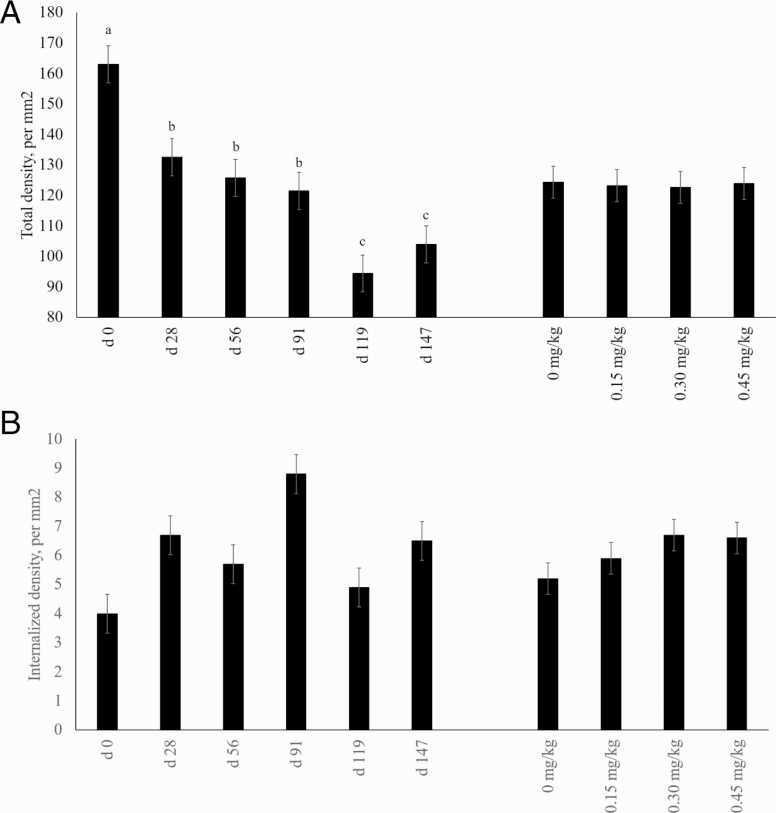
Effects of CrP supplementation on GLUT4 density and characteristics in feedlot steers from longissimus biopsies collected on days 0, 28, 56, 91, 119, and 147 of the feeding trial (*n* = 4). Treatments were 0 mg Cr/kg diet DM, 0.15 mg Cr/kg diet DM (CrP; KemTRACE Chromium, 0.04%, Kemin Industries, Des Moines, IA), 0.30 mg Cr/kg diet DM, and 0.45 mg Cr/kg diet DM. Cross-sections of skeletal muscle samples were stained by immunohistochemistry for presence GLUT4. Panel A depicts total GLUT4 density, and panel B shows the density of internalized GLUT4. Differing superscripts denote means differ by day (*P* ≤ 0.01).

For the total density of GLUT4, there was a tendency for an interaction observed between treatment and day (*P* < 0.05). The density of GLUT4 decreased from days 0 to 147 for all treatments, but the density in samples from the control treatment decreased more than the three treatments receiving Cr (Figure 5). Internalization of GLUT4 was also measured, as shown in Figure 5. No interaction of treatment and day occurred (*P* > 0.05). However, there were treatment effects (*P* < 0.01) and day effects (*P* < 0.001). There was a greater density of internalized GLUT4 on day 147 compared with day 0, and the 0.45 and 0.30 mg/kg treatments had greater densities of internalized GLUT4 than the control treatment. When GLUT4 is found inside the cell, the protein is not able to transport extracellular glucose into the cell for metabolism. The importance of GLUT4 to skeletal muscle homeostasis has been established. Of the known GLUTs, GLUT4 is found most abundantly in insulin sensitive tissues such as skeletal muscle and adipose tissue (Bell et al., 1990). In skeletal muscle and adipose tissue, GLUT4 is translocated to the cellular membrane in response to insulin signaling, allowing for transport of extracellular glucose to the cytosol of the cell (Sano et al., 2003). When GLUT4 is disrupted in murine skeletal muscle, there is a drastic reduction in glucose transport, insulin sensitivity, and glucose tolerance (Zisman et al., 2000). Supplementation with CrP has been shown to increase insulin sensitivity in beef cattle (Kegley et al., 2000; Spears et al., 2012). In rat adipocytes, insulin increased the number of surface GLUT4 (Juhn et al., 1992). This was not seen in the present study. The increase of internalized GLUT4 transporters in the 0.45 mg/kg CrP treatment with no change in insulin levels suggests that CrP supplementation alters the movement of GLUT4 translocation in response to insulin signaling cues.

**Figure 6.**
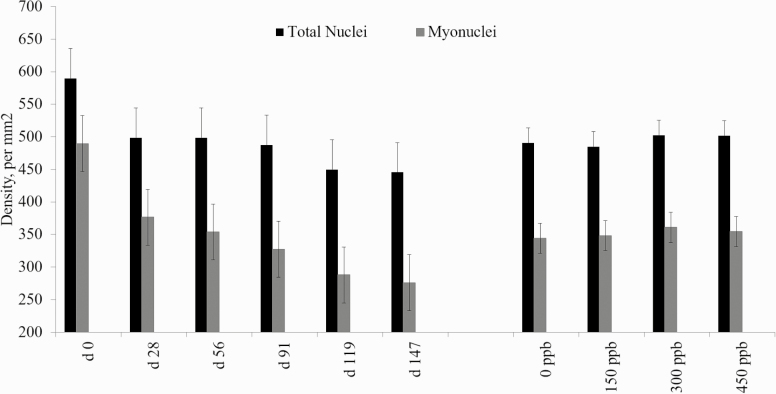
Effects of CrP supplementation on nuclei density in feedlot steers from longissimus biopsies collected on days 0, 28, 56, 91, 119, and 147 of the feeding trial (*n* = 4). Treatments were 0 mg Cr/kg diet DM, 0.15 mg Cr/kg diet DM (CrP; KemTRACE Chromium 0.04%, Kemin Industries, Des Moines, IA), 0.30 mg Cr/kg diet DM, and 0.45 mg Cr/kg diet DM. Cross-sections of skeletal muscle samples were stained by immunohistochemistry for nuclei. Differing superscripts denote means differ by day (*P* < 0.01).

Tendencies for the interaction of treatment and day were seen for the density of both total nuclei and myonuclei, or nuclei associated with the muscle fiber (*P* ≤ 0.08; Figure 6). There was no effect (*P ≥* 0.10) of CrP supplementation for either total nuclei density or for the density of myonuclei. However, for both total nuclei and myonuclei densities, density of nuclei decreased from days 0 to 147. These numbers are reflective of the increase in skeletal muscle fiber cross-sectional area, leading to a dilution of total nuclei as the protein:DNA unit increases (Hosford et al., 2015; Smith et al., 2019). Total nuclei are composed of both nuclei fused into the muscle fiber as well as satellite cells which are next to the muscle fiber to fuse into the cell.

**Figure 7.**
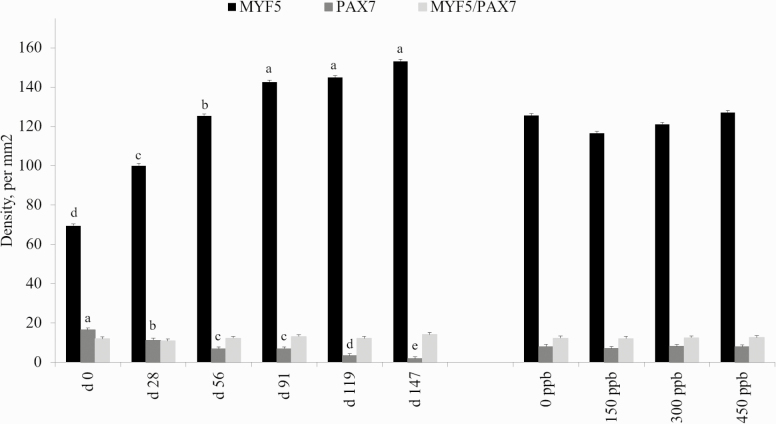
Effects of CrP supplementation on satellite cell population density in feedlot steers from longissimus biopsies collected on days 0, 28, 56, 91, 119, and 147 of the feeding trial (*n* = 4). Treatments were 0 mg Cr/kg diet DM, 0.15 mg Cr/kg diet DM (CrP; KemTRACE Chromium 0.04%, Kemin Industries, Des Moines, IA), 0.30 mg Cr/kg diet DM, and 0.45 mg Cr/kg diet DM. Cross-sections of skeletal muscle samples were stained by immunohistochemistry for satellite cell populations. Differing superscripts denote means within satellite cell population differ by day (*P* < 0.01).

For the satellite cell populations measured (Myf5-positive, PAX7-positive, and dual Myf5/PAX7-positive), no interaction of treatment and day was detected (*P* ≥ 0.10; Figure 7). Additionally, there was no change in the satellite cell population due to Cr supplementation for any of the three populations (*P* > 0.05) analyzed in the present study. A change in the populations of Myf5-positive and PAX7-positive satellite cells over time was observed (*P* < 0.001). In Myf5-positive cells, the density increased from days 0 to 147. Conversely, the density of PAX7-positive cells decreased as days on feed increased. This suggests that there was an increase in the number of proliferative satellite cells coupled with an increase in the fusion of satellite cells into the existing skeletal muscle fibers, as Myf5 is important for satellite cell proliferation while PAX7 is crucial for differentiation of satellite cells (Zammitt et al., 2006; Kuang et al., 2007). Total number of skeletal muscle satellite cells decreases over time (Smith et al., 2019), from 30% of nuclei found in skeletal muscle at birth being associated with satellite cells to 2% to 10% of skeletal muscle nuclei in physiologically mature mice (Cardasis and Cooper, 1975). These results suggest the steers in the present study were still capable of increased skeletal muscle growth, as the number of satellite cells increased over time. The ratio of DNA:protein in skeletal muscle is highly conserved, and Cheek et al. (1971) stated change in the ratio of DNA:protein is determined by DNA and nuclei numbers.

## CONCLUSIONS

Supplementation of CrP to feedlot cattle for 150 d improved ADG, G:F, and HCW. No impact due to treatment on the circulating sera metabolites was observed. Use of CrP in finishing diets up to 0.45 mg Cr/kg of diet DM increases carcass ADG and EBW. The lack of impact on the ratio of carcass ADG:live ADG shows the ADG of carcass and noncarcass components was not impacted differently by CrP. Therefore, these results indicate that the addition of Cr above basal concentration could improve growth performance in feedlot steers, resulting in increased HCW. The combination of no change in total GLUT4 expression with an increase of translocation of GLUT4 from the sarcolemma to the interior of the skeletal muscle fibers due to Cr supplementation imply uptake of glucose by skeletal muscle fibers may become more efficient in animals supplemented CrP, thus requiring fewer glucose transporters on the surface of the fiber. This shift would allow for skeletal muscle fibers to direct nutrients away from glucose transporter synthesis toward increasing body mass, such as skeletal muscle tissue.
